# THE INTERSECTION OF ETHICS AND VULNERABLE ELDERS

**DOI:** 10.1093/geroni/igz038.887

**Published:** 2019-11-08

**Authors:** Pamela B Teaster, Georgia Anetzberger

**Affiliations:** 1 Virginia Tech, Blacksburg, Virginia, United States; 2 Case Western Reserve University, Cleveland, Ohio, United States

## Abstract

Researchers, practitioners and policymakers are daily confronted with multiple and competing situations regarding vulnerable older adults and the complex issues that they face in all aspects of their lives. Challenges can arise in the provision of social services, dispensing justice, conducting research, or addressing legal issues. The purpose of this symposium is to discuss dilemmas that vulnerable older adults and concerned others face by elucidating current and future challenges facing this population, particularly in the realms of compromised health (cognitive impairment); effective status (gender); care arrangements (home and community-based services); and abuse, neglect, and exploitation. Teaster and Anetzberger discuss relevant ethical theories and principles as well as a definition of vulnerability. Santos and Nichols-Hadeed report on ethical issues embedded in vulnerable elders’ cognitive status. Bowland and Halaas highlight the intersection of ethics, gender and vulnerable elders. Niles-Yokum and Beaumaster discuss the nexus of ethics and the provision of home and community based services for vulnerable older adults. Heisler considers vulnerabilities of older adults and ethical challenges when addressing elder abuse. Throughout the papers, we weave the ethical principles of autonomy, beneficence, nonmaleficence, and justice.

